# Functions of TRPs in retinal tissue in physiological and pathological conditions

**DOI:** 10.3389/fnmol.2024.1459083

**Published:** 2024-09-25

**Authors:** Thaianne Hanah Oliveira do Nascimento, Danniel Pereira-Figueiredo, Louise Veroneze, Amanda Alves Nascimento, Francesco De Logu, Romina Nassini, Paula Campello-Costa, Adriana da Cunha Faria-Melibeu, Daniel Souza Monteiro de Araújo, Karin Costa Calaza

**Affiliations:** ^1^Laboratory Neurobiology of the Retina, Department of Neurobiology and Program of Biomedical Sciences, Biology Institute, Fluminense Federal University Niterói, Rio de Janeiro, Brazil; ^2^Laboratory Neurobiology of the Retina, Department of Neurobiology and Program of Neurosciences, Biology Institute, Fluminense Federal University, Rio de Janeiro, Brazil; ^3^Department of Health Sciences, Clinical Pharmacology and Oncology Section, University of Florence, Florence, Italy; ^4^Laboratory of Neuroplasticity, Program of Neurosciences, Department of Neurobiology, Biology Institute, Niteroi, Brazil; ^5^Laboratory of Neurobiology of Development, Program of Neurosciences, Department of Neurobiology, Biology Institute, Niteroi, Brazil

**Keywords:** age-related macular degeneration, glaucoma, retinitis pigmentosa, diabetic retinopathy, TRPs channels

## Abstract

The Transient Receptor Potential (TRP) constitutes a family of channels subdivided into seven subfamilies: Ankyrin (TRPA), Canonical (TRPC), Melastatin (TRPM), Mucolipin (TRPML), no-mechano-potential C (TRPN), Polycystic (TRPP), and Vanilloid (TRPV). Although they are structurally similar to one another, the peculiarities of each subfamily are key to the response to stimuli and the signaling pathway that each one triggers. TRPs are non-selective cation channels, most of which are permeable to Ca^2+^, which is a well-established second messenger that modulates several intracellular signaling pathways and is involved in physiological and pathological conditions in various cell types. TRPs depolarize excitable cells by increasing the influx of Ca^2+^, Na^+^, and other cations. Most TRP families are activated by temperature variations, membrane stretching, or chemical agents and, therefore, are defined as polymodal channels. All TPRs are expressed, at some level, in the central nervous system (CNS) and ocular-related structures, such as the retina and optic nerve (ON), except the TRPP in the ON. TRPC, TRPM, TRPV, and TRPML are found in the retinal pigmented cells, whereas only TRPA1 and TRPM are detected in the uvea. Accordingly, several studies have focused on the search to unravel the role of TRPs in physiological and pathological conditions related to the eyes. Thus, this review aims to shed light on endogenous and exogenous modulators, triggered cell signaling pathways, and localization and roles of each subfamily of TRP channels in physiological and pathological conditions in the retina, optic nerve, and retinal pigmented epithelium of vertebrates.

## 1 Introduction

The visual system is one of the most important senses, contributing to the defense and survival of animals, especially humans, and playing a crucial role in daily activities and complex behaviors (Potier et al., 2018). Visual loss tremendously impacts quality of life, affecting work and leisure activities, such as reading, driving, and watching television. Further, visual deficits/blindness are associated with psychological and social well-being, increasing the probability of developing anxiety and depression (Bradley and Delaffon, 2020).

The external part of the eyeball is composed of the cornea and sclera, followed by the iris, pupil, crystalline, and ciliary body; the posterior part comprises the retina and the choroid (Irsch and Guyton, 2009; Kels et al., 2015). Although all these components are essential to vision, the majority of non-prevented, untreatable visual deficits or blindness results from retinal pathologies (GBD 2019 Blindness and Vision Impairment Collaborators, 2021).

The Transient Receptor Potential (TRP) is an evolutionarily conserved group of channels found in invertebrates, such as the phylum nematodes and arthropods, and in vertebrates, such as fish and mammals (Vriens et al., 2004). They form ion-permeable transmembrane polymodal receptors, classically described in somatosensory neurons but also found in ocular structures, and they are sensitive to different stimulus types (Ma et al., 2022; Samanta et al., 2018). Accordingly, they can be modulated by thermal stimuli, plasma membrane stretching, endogenous/exogenous chemical molecules, fragments of pathogens, and inflammatory signals (Gilliam and Wensel, 2011). The majority of TRPs is permeable to Ca^2+^, a well-known second messenger, which induces several cellular responses (Reinach et al., 2015; Vangeel and Voets, 2019). Currently, seven subfamilies of TRPs are known: Ankyrin (TRPA), Canonical (TRPC), Melastatin (TRPM), Mucolipin (TRPML), NO-mechano-potential C (TRPN), Polycystic (TRPP) and Vanilloid (TRPV) (Samanta et al., 2018). TRPN was not included in this review as it has not been described in the retina.

Since the discovery of these channels, experimental studies seek the structural detail of each subfamily to reach a greater understanding of the modulatory mechanisms, as well as the action of TRP antagonists and agonists in the respective binding sites or through indirect stimulation, to broaden the understanding of the roles of this superfamily in a physiological and pathological context. In the present review, we focus on shedding light on information about the expression, modulation, and physiological function of TRPs and their putative involvement in the main human retinal pathologies.

## 2 Search method

The present review searched for published studies on PubMed, Mendeley, and Scopus platforms, using keywords related to the subject topics. In the introductory topic, the combination of terms used was: “TRP” (AND) “brain” (129 articles), OR “vision” (102 articles), OR “structure” (171 articles). For all other topics, the terms “TRPC OR TRPV OR TRPM OR TRPA1 OR TRPML OR TRPP” were combined (AND) with a specific keyword related to the topic, as follows:

- Structure, functioning, exogenous/endogenous modulators, and signaling pathways: “structure OR modulation OR signaling.”
- Expression and functions in ocular structures: “Retina and retinal OR optic nerve OR pigment epithelium.”
- Ocular diseases: “Macular degeneration.” “Diabetic retinopathy,” “retina,” “methylglyoxal,” “glaucoma,” “intraocular pressure,” “retina” “retinitis pigmentosa,” “rd10,” “rd1.”

For this review, we highlighted studies published in English in the last 5 years that brought new information to the subject up to publication. However, some reviews and data published 10 years ago or more were also included. This was necessary to contextualize information about TRP structure and signal/function in retinal physiology or retinopathies. Supplementary material consists of a diagram of search strategies and the total number of articles for each one.

## 3 TRPs: from modulators to functioning

The first description of the TRP channels was in the *Drosophila melanogaster*, where abnormal retinal responses to high luminosity were documented (Reinach et al., 2015; Samanta et al., 2018). Since then, there has been an increase in research demonstrating the involvement of TRPs in retinal physiology and pathologies that lead to loss or partial impairment of vision in models of glaucoma (de Araújo et al., 2020; Choi et al., 2015; Irnaten et al., 2020; Oda et al., 2020), diabetic retinopathy (Sachdeva et al., 2018), retinitis pigmentosa (Nelidova et al., 2020), and retinal dysfunction promoted in non-ocular diseases such as mucolipidosis type IV (ML-IV) (Grishchuk et al., 2016; Venugopal et al., 2007).

All TRP subfamilies show some structural similarities. They are tetramers, where each monomer consists of six transmembrane domains (TM) (S1-S6) (Bae et al., 2018; Huang et al., 2020; Huffer et al., 2020). S5 and S6 (Huffer et al., 2020) are connected to the p-loop (Bae et al., 2018; Huang et al., 2020), and the three structures comprise the channel selectivity filter (Bae et al., 2018; de Baaij et al., 2014; Huang et al., 2020).

**Figure 1:**
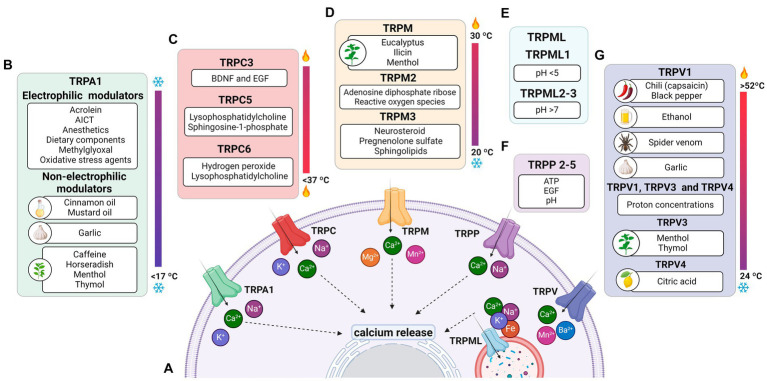
Permeability of TRP receptors and exogenous modulators. **(A)** TRPA1 receptors and TRPCs are permeable to potassium (K^+^), sodium (Na^+^), and calcium (Ca^2+^) ions; TRPM, TRPP and TRPVs receptors are permeable to calcium; the TRPML receptor is permeable to calcium, sodium (Na^+^), iron (Fe), and potassium (K^+^) ions. Since all receptors are permeable to calcium, their activation contributes to an increase in intracellular calcium levels and the release of ions stored in the endoplasmic reticulum. **(B)** Modulators of TRPA1 receptors, including acrylamide-induced cytotoxicity (AICT), dietary components, and oxidative stress agents. **(C)** Regulators of TRPC receptors, including brain-derived neurotrophic factor (BDNF) and epidermal growth factor (EGF). **(D)** Extrinsic modulators of TRPM receptors. **(E)** Extrinsic modulators of TRPML receptors, including adenosine triphosphate (ATP) and changes in acidity/alkalinity (pH). **(F)** Regulators of TRPP receptors. **(G)** Modulators of TRPV receptors.

TRPs are a remarkably diverse group of proteins with several characteristics, which will be highlighted in the present review. Most TRP channels are permeable to monovalent and divalent ions, except for TRPV5 and TRPV6, which are highly ion-selective and have the response amplitude dependent on the extracellular Ca^2+^ concentration (10 and 30 mM, respectively) (Hoenderop et al., 2003; Nilius et al., 2001; Montell, 2005; Voets et al., 2001; Figure 1A). There is also an extension originating right after TM at the end of the N-terminal, called the TRP box, whose activity is related to channel activation and inactivation (Feng, 2017; Huffer et al., 2020).

The C- and N-termini constitute the cytoplasmic region of TRPs (Huffer et al., 2020). At the C-terminus of the TRPC, TRPM, and TRPA1 subfamilies, the coiled-coil (CC) domain appears to play a role in ligand anchoring and assembly of these channels in the lipid bilayer (Feng, 2017; Hilton et al., 2019; Nooren et al., 1999). Furthermore, the N-terminus of TRPA1, TRPC, TRPV, and TRPM exhibits repeats of the ankyrin protein, whose function is related to conformational changes of the ionic pore and target of specific molecules in some TRPs (Feng, 2017; Hilton et al., 2019; Li et al., 2019).

The central pore and the TRP box are highly conserved in the different TRP subfamilies, while S1-S4 show structural modifications, and the C-terminus and N-terminus are quite variable between subfamilies. These differences contribute to establishing specificities and modulatory mechanisms in TRP channels (Huffer et al., 2020; Li et al., 2011). Similarities and differences in the structural components between TRP subfamily members, modulating molecules, as well as the response after activation of these channels, are summarized in Table 1.

**Table 1:** Structural components, modulatory and response mechanism peculiarities among all TRP subfamilies.

TRP	Subcellular location	Structure		Modulation		Effect on the endoplasmic reticulum
		**=**	**≠**	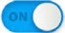	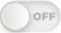	
TRPA	PM	S1–S6; TRP box	AR > 10; CC	Ca^2+^; Ca^2+^/CaM; PKA/PKC; PIP_2_	??	(+) calcium release
TRPC	PM	S1–S6; TRP box	AR < 10; helix and pre helix loop-S1; CC	Ca^2+^; Ca^2+^/CaM; CIRB; STIM/Orai; PIP_2_; PLC/IP_3_/DAG	Ca^2+^/CaM	(+) calcium release
TRPM	PM	S1–S6; CC; TRP box	MHR; enzymatic domain; CC	Ca^2+^ (−); CaM	PIP_2_	(+) calcium release
TRPML	Lysosomes	S1–S6	N-glycosylated loop	Ca^2+^; PKC; PI(3,5)P_2_	PKA; PIP_2_; PI(3,5)P_2_	(+) calcium release
TRPP	PM	S1–S6; TRP box	AR > 10; polycystin loop	Ca^2+^	PIP_2_	(−) calcium release
TRPV	PM	S1–S6; TRP box	AR < 10; helix pre-S1	Ca^2+^ (+); CaM; PKA/PKC; PIP_2_	PIP_2_	(+) calcium release

TRP structural conformation and response to Ca^2+^ release. **=**: similarity; **≠**: difference; 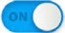: activation; 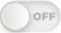: deactivation; PM: Plasmatic membrane; AR: Ankyrin; <: smaller; >: bigger; CC: coiled-coil; Melastatin homology regions: MHR; Calcium/calmodulin: Ca^2+^/CaM; Protein kinase A: PKA; Protein Kinase C: PKC; phosphatidylinositol 4,5-bisphosphate: PIP_2_; inositol (1,4,5)-trisphosphate receptor binding: CIRB; Stromal interaction molecule/activated calcium channel protein: STIM/Orai; Phospholipase C: PCL; Inositol 1,4,5-trifosfato: IP_3_; Diacylglycerol: DAG; Calmodulin: CaM; Phosphatidylinositol 3,5-bisphosphate: PI(3,5)P2; ??: unknown; (+): increase; (−): reduction; Calcium: Ca^2+^; Highly selective to calcium: Ca^2+^(+); Calcium impermeable: Ca^2+^(−).

### 3.1 TRPA1

TRPA1 is a cation-permeable channel, especially to Ca^2+^ (Talavera et al., 2020; Figure 1A). This receptor is mechanical-, thermo-, and chemo-sensitive and can be activated by exogenous and endogenous agents (Kádková et al., 2017). There are binding sites for channel-modulating substances in TRPA1 transmembrane domains, which also appear important for voltage-sensitivity and -dependence. Marsakova and collaborators suggest a binding site for Ca^2+^ in the external region of the TRPA1 protein and that the S1-S2 domains are physically coupled to the gating regions of the channel (Marsakova et al., 2017). On the other hand, recent experiments by Zhao et al. (2020) suggest that Ca^2+^-dependent modulations appear to be concentrated in S2–S3 (Zhao et al., 2020). The size of the NH2-terminal is equivalent to all the rest of the protein, consisting of 14–18 repetitions of ankyrin, a characteristic that gave TRPA1 its name (Meents et al., 2017; Nilius et al., 2011). In addition to being a binding site for several molecules, such as cytosolic Ca^2+^, it also appears to participate in thermal and chemical responsiveness (Cordero-Morales et al., 2011; Jabba et al., 2014).

The N-terminal region can regulate and integrate stimuli (Zayats et al., 2013; Hynkova et al., 2016). Many possible forms of channel modulation involving the N-terminal region have been described: (1) irritating agonists and electrophiles (isocyanates, cinnamaldehyde, garlic diallyl disulfide, acrolein) can chemically react with primary amines and cysteines (Bahia et al., 2016; Suo et al., 2020) inducing channel opening (Hinman et al., 2006; Macpherson et al., 2007); (2) direct calcium binding participating in desensitization (Cordero-Morales et al., 2011); (3) direct interaction with proteins such as FGFR2, which inhibit TRPA1 (Berrout et al., 2017); (4) phosphorylation, for example by protein kinase A (PKA) or protein kinase C (PKC), which can increase levels of the channel on the plasma membrane. The C-terminal termination also has Ca^2+^ binding sites but seems more associated with voltage-dependent responses (Sura et al., 2012). A summary of the stimuli of TRP is shown in Table 2.

**Table 2:** Modulators of TRP channel activity.

TRP	Temp	Mechanosensitivity		Endogenous ligands		Exogenous ligands	
	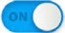	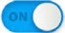	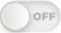	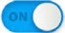	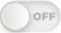	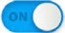	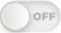
TRPA	<17°C	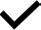	–	NO, H_2_O_2_, Methylglyoxal, ROS, RNS, RCS, 4-HNE, p+	Resolvin	Caffeine, alicin, isoflurane lidocaine	Caffeine, nicotine, menthol
TRPC	<37°C	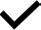	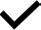	Lisofosfatidilcolina, H_2_O_2_	Cholesterol, Ca^2+^/CaM, Na^+^, Mg^2+^, Ca^2+^	Hiperforin	Pyrazoles
TRPM	20°C – 30°C	??	–	Mg^2+^, ADPR, ROS, sphingolipids, pregnenolone sulfate	Zn^+^, AMP, pH < 7, Mg^2+^, Cholesterol, ATP	Allicin, menthol, eucalyptus	2-APB, carvacrol
TRPML	??	??	??	pH <5 pH >7	**–**	ML-SA1	**–**
TRPP	??	??	??	pH, EGF, ATP	??	**–**	**–**
TRPV	24°C - >52°C	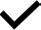	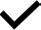	Cholesterol, p+, ATP	Mg^2+^	Capsaicin, DkTx, menthol	Capsazepine

Summary of several types of TRP channel agonists and antagonists. Temp: temperature; 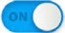: activation; 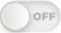: deactivation; <: smallest; >: biggest; 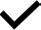: reported as modulator; −: no modulatory effect; ??: unknown; 4-HNE: 4-hydroxynonenal; p+: proton; 2-APB = 2-aminoethoxydiphenyl borate; ML-SA1: Mucolipin synthetic agonist 1.

It is known that the activation of all TRPs promotes the entry of Ca^2+^ into the cell, which, in turn, triggers the activation of biochemical pathways, such as the calcium-dependent-phospholipase C (PLC) pathway (Kouchi et al., 2004), which hydrolyzes phosphatidylinositol 4,5-bisphosphate (PIP_2_) into inositol 1,4,5-trifosfato (IP_3_) and diacylglycerol (DAG) (Wang et al., 2020) and then IP_3_ binds to IP_3_ receptors located in the endoplasmic reticulum (ER), promoting a transient increase in cytosolic Ca^2+^ (Curcic et al., 2019; Mori et al., 2015; Figure 1A). PIP_2_ is a major modulator of TRP channels, and it can sometimes inhibit or activate, as will be discussed later (Pumroy et al., 2020). Usually, one of the interaction sites with PIP_2_ is located at the N-terminus of TRPs, and in the case of TRPA1, it is also located at the C-terminus (Macikova et al., 2019).

TRPA1 can be modulated by a huge variety of chemical compounds, divided into electrophilic and non-electrophilic modulators, according to the ability of each group to interact covalently or noncovalently, respectively (Talavera et al., 2020; Figure 1B). Among the former group, there are dietary components such as cinnamaldehyde, allicin (Bandell et al., 2004), isothiocyanates (Bandell et al., 2004), thymol, methyl salicylate; anesthetics as isoflurane, lidocaine, and propofol (Matta et al., 2008); and oxidative stress agents such as nitric oxide (NO), hydrogen peroxide (H_2_O_2_), and methylglyoxal (MG) (Cao et al., 2012; Meents et al., 2017), although reactive oxygen (ROS), nitrogen (RNS), and carbonyl (RCS) species are all, in general, potential activators (Bessac and Jordt, 2008; Nilius et al., 2011). In the context of oxidative stress, the product of lipid peroxidation, 4-hydroxynonenal (4-HNE), is also a potent activator of TRPA1 (Trevisani et al., 2007). The non-electrophilic group includes some commonly consumed components, such as menthol (Karashima et al., 2009), thymol (Lee et al., 2008), carvacrol (Xu et al., 2006), nicotine, caffeine, and Δ^9^–tetrahydrocannabinol; medication like nifedipine and nonsteroidal anti-inflammatory drugs (Jordt et al., 2004; Nassini et al., 2015; Talavera et al., 2009); and protons (Nilius et al., 2011). Interestingly, some ligands have a bimodal effect, activating the channel in low concentrations while inhibiting it at higher doses, as it is the case of nicotine and menthol (Karashima et al., 2009; Talavera et al., 2009). Furthermore, there are also components with species-specific effects when it comes to modulation of the channel; for example, caffeine can activate TRPA1 in mice but suppresses it in humans (Nagatomo and Kubo, 2008).

Ca^2+^ is another important modulator of TRPA1 activity. It interacts intracellularly first to activate and then increases the response to other activators, followed by an inactivation process (Sura et al., 2012). Both potentiation and inactivation appear to be mediated by independent processes (Wang et al., 2008). In addition, there is evidence that modulation by Ca^2+^ is dependent on the direct binding of calmodulin to the channel, which affects both the activation and inactivation steps (Hasan et al., 2017), although the direct action of Ca^2+^ on human TRPA1 has also been demonstrated by calmodulin-independent mechanisms (Moparthi et al., 2020).

Protons can also activate TRPA1 in the context of acidosis, a common event in ischemia, for example. Intracellular protons are more commonly accepted as modulators, while extracellular activation is species specific (de la Roche et al., 2013). Additionally, phosphorylation sites for kinases, such as PKA and cyclin-dependent kinase 5 (Cdk5), have been identified and correlated with the capacity to promote sensitization of the channel, enabling increased responsiveness to agonist stimulation (Meents et al., 2017; Hall et al., 2018). As demonstrated recently, PKC can also sensitize the channel (McMillan et al., 2021), but data concerning more direct interaction needs further analysis.

### 3.2 TRPC

TRPC 1–7 are classified as non-selective channels, permeable to Na^+^, K^+^, and Ca^2+^ (DeHaven et al., 2009; Figure 1A). TRPCs are abundantly expressed in immune cells, endothelium, heart, striated muscle, pancreas, kidneys, placenta, retina, pituitary gland, cerebellum, limbic system, and substantia nigra (Wang et al., 2020; Warren et al., 2006). Only TRPC2 is not expressed in human cells (de Souza and Ambudkar, 2014).

The carboxy-terminal region commonly comprises domains that bind Ca^2+^/CaM complex and inositol (1,4,5)-trisphosphate receptor binding (CIRB), and other molecules, such as trimeric G proteins (Friedlova et al., 2010; Wang et al., 2010; Wang et al., 2020). Although the mechanisms of TRPC activation are not yet well described (Wang et al., 2020), it is known that they are channels dependent on PLC, either activated by G protein-coupled receptors or tyrosine kinase receptors (Feng, 2017; Li et al., 2019; Wang et al., 2020; Zhu, 2005).

In response to PLC signaling, there is an elevation in calcium levels in the cytoplasm, with a consequent depletion of Ca^2+^ stores in the ER, resulting in activation of TRPC, which promotes robust and sustained Ca^2+^ entry by the channel (Curcic et al., 2019; Hofmann et al., 1999; Mori et al., 2015; Zhu, 2005).

Therefore, it is proposed that TRPC represent store-operated Ca^2+^ entry channels (SOCE), as they play a key role in increasing cytoplasmic Ca^2+^ (DeHaven et al., 2009; Feng, 2017; Li et al., 2019). Furthermore, intracellular Ca^2+^ moderately potentiates TRPC4 responses and considerably TRPC5, possibly due to a longer opening time of both ionic pores (Blair et al., 2009; Mori et al., 2015). Although TRPC1 alone functions as a SOCE in human adult retinal pigment epithelial-19 (ARPE-19) cells (Bollimuntha et al., 2005), TRPC3 and 4 only function as SOCE when co-expressed with TRPC1 (de Souza and Ambudkar, 2014). It has already been demonstrated that these TRPCs function as a SOCE channel in cultured Müller glia and 129/SvImJ mice (Molnár et al., 2016; Da Silva et al., 2008).

Physical stimuli have been linked to the activation of TRPCs. TRPC1, C5, and C6 are characterized as mechanosensitive receptors (Choi et al., 2015; Irnaten et al., 2020), while TRPC5 is also stimulated by temperatures below 37°C (Oda et al., 2020; Figure 1C).

TRPC1, 4, and 5 are activated directly by phospholipase C (Duan et al., 2018; Chen et al., 2020). Both TRPC3 and C5 can be stimulated by IP_3_, while C3, 6, and 7 exhibit varying sensitivity in the presence of PIP_2_ (Wang et al., 2020). TRPC2, 3, 6, and 7 are directly stimulated by DAG (Fan et al., 2018; Li et al., 2019; Wang et al., 2020), while TRPC may not be stimulated by DAG in ipRGCs culture (Graham et al., 2008). Although TRPC4 and C5 are not activated by DAG, they may become sensitive to the molecule and its derivatives (Curcic et al., 2019; Storch et al., 2017). This sensitivity change in TRPC4-5 occurs while PKC activity is inhibited since the kinase deactivates these TRPCs as well as PIP_2_ (Storch et al., 2017).

### 3.3 TRPM

The TRPM family consists of eight members (TRPM1–TRPM8), expressed in cells distributed in different tissues, such as the retina, kidney, pancreas, prostate, heart, intestine, and immune system (Huang et al., 2020; Sawamura et al., 2017). In the nervous system, TRPM is detected in neurons and glial cells (astrocytes, oligodendrocytes, and microglia) (Sawamura et al., 2017).

TRPM1, TRPM2, TRPM3, TRPM6, TRPM7, and TRPM8 share similarities in generating responses through Ca^2+^ influx (Bae et al., 2018), while TRPM4 and TRPM5 are voltage-dependent and Ca^2+^ impermeable (Huang et al., 2020; Samanta et al., 2018). TRPMs are also permeable to Mg^2+^ and Mn^2+^; Mg^2+^ negatively modulates the TRPM1 channel expressed in the photoreceptor (Jimenez et al., 2020; Figure 1A). In addition, TRPM5 is activated by intracellular Ca^2+^ with greater sensitivity than TRPM4 (Ruan et al., 2021; Zierler et al., 2017).

The TRPM has particular structural characteristics that are distinct from other TRPs. They are the receptors with the highest amount of amino acid residues in the cytosolic region and do not have the N-terminal ankyrin repeats (Huang et al., 2020; Kraft and Harteneck, 2005; Samanta et al., 2018). The N-terminus comprises four melastatin homology regions (MHRs), while the C-terminus exposes two domains (Chen et al., 2019; Guo et al., 2017).

Furthermore, the cytoplasmic domain may show differences between members of the TRPM family (Huang et al., 2020; Jirku et al., 2015; Zierler et al., 2017). For example, most members of the TRPM family, including TRPM1, exhibit binding sites in both the transmembrane domain and the N-terminus for interaction with PIP_2_ (Huang et al., 2020; Jirku et al., 2015; Zierler et al., 2017). The C-terminus of TRPM2, 6, and 7 have enzymatic domains (Guo et al., 2017; Jirku et al., 2015; Samanta et al., 2018). The enzymatic domains reported in experimental data at the C-terminus of TRPM6 and 7 are comprised of alpha kinases, a class of kinases that preferentially phosphorylate on the alpha helix of the target molecule. These kinases can induce positive modulatory functions related to Mg^2+^ (Cabezas-Bratesco et al., 2015; Cai et al., 2017); the ion also plays a negative role in the activation of TRPM1 in retinal cells (Rampino and Nawy, 2011). Conditions of increased ROS production and oxidative stress induce ADPR release and TRPM2 activation (Tóth et al., 2014; Huang et al., 2019; Huang et al., 2018; Figure 1D).

It has been described that at the N-terminus of TRPM2, there is an isoleucine glutamine (IQ) domain, reported as the main binding site for the Ca^2+^/CaM complex (Du et al., 2009; Kim et al., 2008; Syed Mortadza et al., 2015). Experiments involving mutations in amino acids of the IQ motif resulted in inactivity of TRPM2 when the channel was stimulated by increased intracellular Ca^2+^ or ADPR, suggesting that the Ca^2+^/CaM complex plays a crucial role in the activation of TRPM2 (Du et al., 2009).

TRPMs play a variety of physiological and biochemical roles in health and disease (Jimenez et al., 2020). For example, TRPM1 plays a key role in the response of retinal ON-bipolar cells (Rampino and Nawy, 2011; Wong et al., 2019); TRPM2 is an oxidative stress sensor in human monocytic cells (Belrose et al., 2012; Sawamura et al., 2017; Wehrhahn et al., 2010); TRPM3, TRPM4, and TRPM5 are involved in the recognition of high temperatures, and TRPM8 in cold (Talavera et al., 2005; Vriens et al., 2011; Figure 1D). In addition, this channel has also been classically described to be activated by organic compounds such as menthol, eucalyptus, and ilicin (Jimenez et al., 2020; Kraft and Harteneck, 2005; Venkatachalam and Montell, 2007; Figure 1D). TRPM6 and TRPM7 play a role in Mg^2+^ homeostasis (Chen et al., 2019; Jin et al., 2008; Wong et al., 2019).

TRPMs are also stimulated by a variety of endogenous agents: TRPM2 activation occurs by ADPR and its analogs, in addition to reactive oxygen species (ROS) (Jimenez et al., 2020; Kraft and Harteneck, 2005; Montell, 2005); TRPM3 is activated by sphingolipids, a class of membrane lipids (Jimenez et al., 2020; Kraft and Harteneck, 2005), and neurosteroid pregnenolone sulfate (PS) during the development of mice retinal cells (Samanta et al., 2018; Shiels, 2020); both TRPM4 and TRPM5 are activated by increased intracellular Ca^2+^ and the co-expression of TRPM6 and TRPM7 turn them responsive to intracellular Mg^2+^ concentrations ranging from 0.3 to 1 mM (Ferioli et al., 2017; Montell, 2005; Figure 1A). TRPM5 is positively stimulated by extracellular adenosine triphosphate (ATP) (Hofmann et al., 2003; Jimenez et al., 2020), and there is evidence that TRPM7 shows the same modulation (Jimenez et al., 2020; Kraft and Harteneck, 2005; Montell, 2005).

The TRPM family is blocked by a variety of molecules and microenvironmental conditions (Jimenez et al., 2020). TRPM1 is down-regulated by Zn^2+^ (Lambert et al., 2011); TRPM2 is inactivated by AMP and pH < 7 (Du et al., 2009; Lange et al., 2008); TRPM3 is blocked by Mg^2+^ and cholesterol (Lambert et al., 2011); TRPM4 is inactivated by cytosolic ATP (Guo et al., 2017); TRPM5 is blocked by acidic pH (D. Liu et al., 2005); and TRPM6 and TRPM7 are blocked by Mg^2+^ (Ferioli et al., 2017). TRPM6 dependent-P2X4 inhibition has also been demonstrated (de Baaij et al., 2014), while the inactivation of TRPM8 occurs by the activity of bradykinin and histamine signaling during inflammatory processes (Zhang et al., 2012a,b).

### 3.4 TRPML/TRPP

TRPML (1–3) are expressed in kidneys, lungs, endocrine tissues, heart, and brain (Samanta et al., 2018; Wang et al., 2014). TRPML1 is the best known and studied within the subfamily, while the other two channels have not yet been reported to impact the health or pathologies of the organism. Therefore, in relation to permeability, it is only known that TRPML1 is permeable to Ca^2+^, Na^+^, K^+^, Fe^2+^, and Zn^2+^ (LaPlante et al., 2002; LaPlante et al., 2004; Zhang et al., 2018; Pedersen et al., 2005; Figure 1A).

TRPML channel proteins have fewer amino acids than other TRPs, making them smaller, with no ankyrin repeats, with an incomplete TRP box, and. Binding sites for PKC between S1 and S2 (Samanta et al., 2018; Spix et al., 2020; Wang et al., 2014).

Moreover, TRPML1 participates in phagocytosis and protein trafficking (LaPlante et al., 2002; Spix et al., 2020). Importantly, TRPML1 mutations, or deletions in the channel, cause ML-IV with serious deleterious ophthalmologic symptoms, including strabismus, myopia, light sensitivity, and retinal degeneration (Smith et al., 2002; Grishchuk et al., 2016).

As illustrated in Figure 1E, variations of pH activate these channels: TRPML1 by pH <5 and TRPML2 and 3 by pH >7 (Spix et al., 2020). Similar to most TRPs, TRPML is mainly up-regulated, but in some cases down-regulated, by phosphatidylinositol 3,5-bisphosphate [PI(3,5)P_2_], found in lysosomes (Dayam et al., 2015; Zhang et al., 2012a,b). The engulfment of potentially harmful particles by phagocytic cells, for example, induces the formation of PI(3,5)P_2_, which acts directly by stimulating TRPML1, expressed in the lumen of organelle, to evoke Ca^2+^ release (Dayam et al., 2015; Zhang et al., 2012a,b; Wang et al., 2014).

PKA can inhibit TRPML1, and when the channel is expressed in the plasma membrane, it is inactivated by low extracellular pH and PIP_2_. PIP_2_ inactivates TRPML1 even in the presence of the agonist, while phospholipid hydrolysis has been shown to activate the channel in HEK293 (Venkatachalam et al., 2015; Wang et al., 2014; Zhang et al., 2012a,b).

Similar to TRPML channels, there is still little information about the permeability of the TRPP subfamily, with only Ca^2+^permeability known for TRPP2-5 and, specifically, for P2 being permeable to Na^+^ as well (Pedersen et al., 2005).

Due to structural differences from TRP and other TRPPs, TRPP1 was considered to form another family known as polycystin1 (PC1), which is a non-functional channel. TRPPs are channels found in various tissues, such as the nervous system, heart, placenta, intestine, and pancreas (Hofherr and Köttgen, 2011; Samanta et al., 2018).

TRPPs have only one specific structural feature, an extensive loop called polycystin, which projects to the cytoplasm and is found in the middle of S1-S2. This is similar to TRPML, whereas in TRPP this structure is related to the stabilization of the channels in the plasma membrane (Hofherr and Köttgen, 2011; Samanta et al., 2018).

Wegierski’s group proposes that Ca^2+^ release in ER is reduced in the presence of TRPP2, and the absence of the channel promotes a huge outflow of Ca^2+^ from the organelle (Wegierski et al., 2009). Similar to TRPC, TRPP2 plays a role in balancing ER Ca^2+^ concentrations (Hofherr and Köttgen, 2011). On the other hand, Zheng and colleagues suggest that PIP_2_ has an inhibitory role in TRPP2-3 (Zheng et al., 2018). Further studies are needed for TRPP to elucidate the role of modulators. It has been mentioned that pH (Huang et al., 2006; Ng et al., 2019), EGF (Zhang et al., 2013) and ATP (Cantero Mdel et al., 2015) are activators and that natural exogenous ligands are unknown (Figure 1F).

### 3.5 TRPV

The TRPV subfamily comprises six channels (1–6), permeable to Ca^2+^, Ba^2+^, Mn^2+^ (Owsianik et al., 2006; Figure 1A). These channels are commonly expressed in the dorsal root, trigeminal, vagus nerves (Samanta et al., 2018; Seebohm and Schreiber, 2021), retina, and optic nerve (Choi et al., 2015; Leonelli et al., 2010; Leonelli et al., 2009), but also in other tissues, smooth muscle, digestive system, kidneys, and pancreas (Samanta et al., 2018; Seebohm and Schreiber, 2021).

TRPV has a smaller helix that precedes S1 and six ankyrin repeats that recognize modulatory molecules (Garcia-Elias et al., 2015; Pumroy et al., 2020). In the amino and carboxy terminal regions, there are binding sites to CaM, ATP (Phelps et al., 2010; Seebohm and Schreiber, 2021), PKA and PKC (Mamenko et al., 2013; Seebohm and Schreiber, 2021) that can regulate the activity of these channels (Mamenko et al., 2013; Niemeyer, 2005; Phelps et al., 2010). In particular, PIP_2_ has a controversial function in TRPs (Pumroy et al., 2020). Low levels of the phospholipid inhibit TRPV1, as well as partially inactivate V2, and completely V6, under the same conditions (Cao et al., 2013; Lukacs et al., 2007; Mercado et al., 2010). PIP_2_ can stimulate V1 in high concentrations of TRPV1 agonists (Lukacs et al., 2007); the reduction of PIP_2_ activates V3, and in V4, the phospholipid stimulates the channel during response to heat and decreases resistance to muscle stretch; V5 is activated by elevated PIP_2_ levels (Doerner et al., 2011; Garcia-Elias et al., 2013; Lee et al., 2005), PIP_2_ also has the potential to enhance TRPV3 functionality and regulate TRPV5 inhibitory responses to Mg^2+^, highlighting the complexity and controversial activity of inositol phospholipid (Lee et al., 2005; Pumroy et al., 2020; Seebohm and Schreiber, 2021).

Originally, most TRPV channels respond to thermal stimuli: TRPV1 (> 42°C), TRPV2 (> 52°C), TRPV3 (31–39°C), and TRPV4 (24–33°C). TRPV5 and TRPV6 are not heat sensors but playa relevant role in Ca^2+^ regulation, given their high ionic selectivity. The TRPV subfamily responds to elevated osmotic pressure, as seen in a glaucoma model, by activating TRPV1 (Sappington and Calkins, 2008). Additionally, it activates TRPV2 and TRPV4 (Muraki et al., 2003; Vriens et al., 2004) while inactivating TRPV5 and TRPV6 (Gangwar et al., 2017; Lewis et al., 2011; Figure 1E).

Capsaicin was the first molecule described to stimulate TRPV1 (Caterina et al., 1997; Figure 1E). Furthermore, V1 was reported to be activated by ethanol in sensory nerves, while garlic extract activated V1 channels expressed in Xenopus oocytes. The amplitude of the TRPV3 response depends on the increase in menthol concentration; the channel is also activated by thymol. TRPV4 has been reported to be stimulated by citric acid solution (Buday et al., 2017; Macpherson et al., 2005; Nicoletti et al., 2008; Vogt-Eisele et al., 2007). TRPV1 and TRPV4 detect high concentrations of protons, while TRPV3 responds to low concentrations (Seebohm and Schreiber, 2021). Furthermore, cholesterol acts on TRPV1 and TRPV3 (Klein et al., 2014; Picazo-Juárez et al., 2011).

It is possible that activation of V1-V2 promotes Ca^2+^ entry into the cell, which, in turn, triggers activation of a type of PLC sensitive to calcium (Lukacs et al., 2007; Mercado et al., 2010). This hydrolyzes PIP_2_ into IP_3_ and DAG (Wang et al., 2020). Then, IP_3_ binds to IP_3_ receptors located in the ER, promoting a transient increase of cytosolic Ca^2+^ (Curcic et al., 2019; Mori et al., 2015).

Mercado and collaborators suggest that PIP_2_ binds directly to active TRPV2, and the hydrolysis of the phospholipid in the plasma membrane is dependent on the entry of Ca^2+^ into the channel; this negative modulation of PIP_2_ levels attenuates the responses triggered by TRPV2 (Mercado et al., 2010). Interestingly, the Ca^2+^/CaM complex did not bind to the channel’s ankyrin repeats, contrary to what was observed in the other TRPVs. Mercado et al. (2010) have described that this complex might not be functional and therefore would have no role in TRPV2 desensitization (Mercado et al., 2010; Rosenbaum et al., 2004; Strotmann et al., 2003). It was later reported that deletion of the Ca^2+^/CaM complex binding site from the N-terminus prevented interaction with IP_3_ receptors, and although TRPV4 still triggered Ca^2+^ influx, no current amplification was observed in the presence of ATP. These results indicate that the channel activity does not depend on the Ca^2+^/CaM complex, but the responses are potentiated by communication with the protein binding site (Garcia-Elias et al., 2008; Niemeyer, 2005).

TRPV4 current can be significantly enhanced by cytoplasmic ATP. Interestingly, this modulation only occurs when the channel is activated by osmotic changes, but not by temperature (Fernandes et al., 2008).

## 4 TRPs expression and functions in the healthy retina and disease conditions

**Figure 2:**
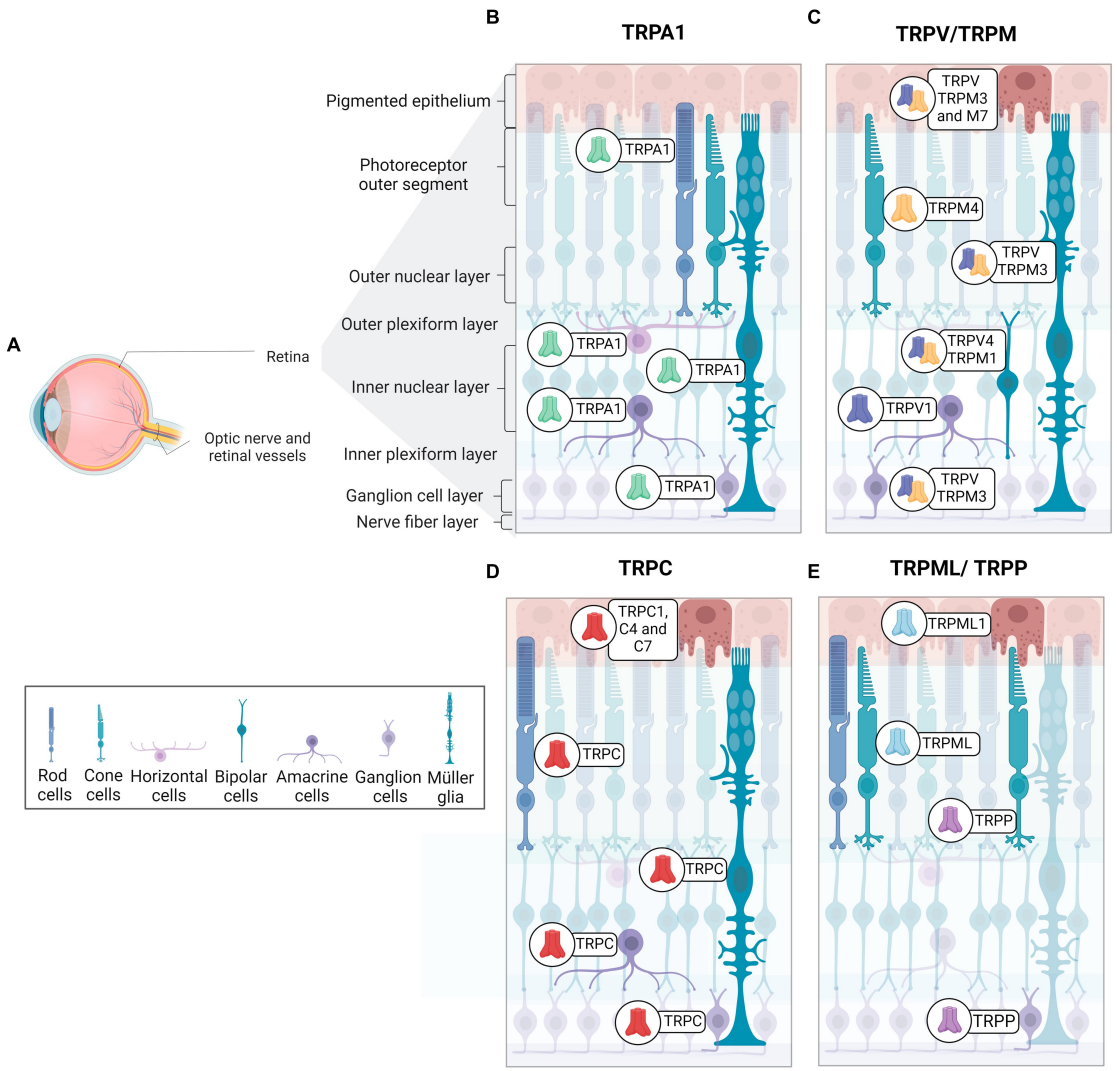
Distribution and expression of TRP receptors in retinal cells. **(A)** Schematic representation of a radial section of the eye indicating the location of the retina and optic nerve. **(B)** TRPA1 receptors are present in photoreceptors, horizontal cells, Müller glia, amacrine cells, and ganglion cells. **(C)** TRPM receptors are identified as follow in epithelial cells (TRPM2, TRPM3 and TRPM7), Müller glia and ganglion cells (TRPM3), cone-type photoreceptors (TRPM4), bipolar cells (TRPM1). **(D)** TRPC receptors are distributed in retinal pigmented epithelium cells (TRPC1, TRPC4, TRPC7), rod-type photoreceptors, Müller glia (TRPC1, TRPC5, TRPC6), bipolar cells (TRPC6), amacrine cells (TRPC5, TRPC6), and ganglion cells (TRPC5, TRPC6). **(E)**TRPML1 receptors are expressed in retinal pigment cells and photoreceptors. TRPP2 receptors are found in cone photoreceptors and ganglion cells. TRPV receptors are observed in pigment epithelium (TRPV1-TRPV4), Müller glia (TRPV1, TRPV4), amacrine cells (TRPV1), bipolar cells (TRPV4) and ganglion cells (TRPV1).

The retina is organized into three cellular layers interspersed with two plexiform layers (Wässle, 2004). The cell layers include the ganglion cell layer (GCL), composed by cell bodies of ganglion cells and displaced amacrine cells; the inner nuclear layer (INL), constituted of bipolar, amacrine, horizontal cells and Müller cells; and the outer nuclear layer (ONL), composed by photoreceptor cells (rods and cones) (Masland, 2001). Synaptic contacts in the retina occur in the inner plexiform layer (IPL), located between the GCL and INL, and the outer plexiform layer (OPL) between INL and ONL. Therefore, the IPL contains synapses of ganglion cells, amacrine, and bipolar cells, while the OPL presents synapses between photoreceptors and bipolar and horizontal cells (Wässle, 2004). Ganglion cell axons form the optic nerve, which connects the retina to brain visual areas. The retinal pigmented epithelium (RPE) is the outermost layer of the retina, adjacent to the choroid, and is important for the physiology of the healthy retina (Wimmers and Strauss, 2007). The RPE controls the trafficking of substances by removing debris from degenerated photoreceptors, ion flux, and releases protective signals, among other functions. Pigmentation of epithelial cells plays a crucial role in preventing light scattering, important to a better resolution of image formation (Wimmers and Strauss, 2007). TRPs family members are widely distributed in the retina (Figure 2).

Araújo and collaborators detected TRPA1 in amacrine, horizontal, ganglion cells, Müller glia, and photoreceptors in mouse and human retinas (de Araújo et al., 2020; Figure 2B). Although TRPA1 seems to play a crucial role in the cell death promoted by ischemia (de Araújo et al., 2020; Araújo et al., 2017), the function in the healthy retina is still undescribed. The same group also demonstrated the presence of TRPA1 in the chick retina, and its role in cell death induced by acute ischemia (Araújo et al., 2017). Table 3 summarizes the expression of TRPs in regions of retina.

**Table 3:** Expression of all TRPs in the retinal layers and optic nerve

TRP	Retina					Optic nerve	Retinal pigmented epithelium
	GCL	INL	IPL	ONL	OPL		
TRPA	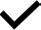	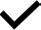	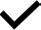	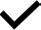	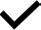	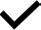	??
TRPC	C5, C6	C1, C6, C7	–	–	C6, C7	C1-C6	C1, C4, C7
TRPM	M3, M4	M1, M3	M1	M4	M1, M3	M1-M4, M6-M7	M3, M7
TRPML	??	??	??	LM1	??	LM1	LM1
TRPP	P2	P2	P2	P2	P2	P1-P3	??
TRPV	V1,V2	V1	V1,V2	V1,V2	V1,V2, V4	V2, V4	V1–V4

Summary of TRP channel expression in ocular regions. 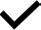: reported location; ?? = unknown; −: not present.

TRPC5 channels are expressed in Müller glial cells, amacrine cells, displaced amacrine, and ganglion cells of C57BL/6 mice retina (Oda et al., 2020; Wang et al., 2010; Figure 2C). Müller glia responds to various stimuli, such as mechanical stimuli, and indirectly to light, with an intense increase in cytoplasmic Ca^2+^ (Da Silva et al., 2008). Müller glia *in vitro* express TRPC1 and C6 mRNA and responds to DAG and its analog with potentiated Ca^2+^ current, suggesting a pivotal role for TRPC as SOCE channels in this cell (Molnár et al., 2016; Da Silva et al., 2008). In agreement, TRPC1/3 knockouts (Molnár et al., 2016) and specific pharmacological blockers of TRPC promote a marked decrease in intracellular Ca^2+^ currents in Müller cells, supporting the hypothesis that SOCE is a TRPC channel (Molnár et al., 2016; Da Silva et al., 2008).

TRPC6 actively participates in phototransduction in intrinsically photosensitive RGCs (ipRGCs), but C3 and C7 may have a role in the response mechanism of ipRGCs, a small group of ganglion cells that contain melanopsin and respond to light with depolarization and Ca^2+^ influx (Berson, 2007; Perez-Leighton et al., 2011). ipRGCs are involved with contrast control, day-night cycle, pupil diameter modulation, and humor (Berson, 2007; Contreras et al., 2021; Sonoda et al., 2018).

TRPC6 and C7 are expressed in ganglion cells, INL, and OPL (Perez-Leighton et al., 2011; Warren et al., 2006), and TRPC6 also occurs in ipRGCs (Perez-Leighton et al., 2011; Figure 2C). TRPC3, 6, and 7 channels are activated by high light intensity (Sonoda et al., 2018).

Many studies have examined phototransduction in ipRGCs. It is suggested that Gq protein activation induces PLC/PIP_2_ hydrolysis and generates IP_3_ and DAG, which stimulate TRPC and increase intracellular Ca^2+^ in response to light stimulation (Contreras et al., 2021; Bailes and Lucas, 2010; Graham et al., 2008). However, despite data proposing that neither DAG nor its derivatives were able to trigger action potentials in ipRGCs in an attempt to activate TRPC channels (Graham et al., 2008), other results have shown that phototransduction was blocked pharmacologically through the use of inhibitors for TRPC, which also act on IP_3_ (Bailes and Lucas, 2010; Sekaran et al., 2007).

TRPC4 occurs in human retinal microvascular endothelial cells (HRMECs) and it seems to be related to angiogenesis. The vascular endothelial growth factor (VEGF), the main modulator of the growth of new blood vessels, lost its angiogenic property in TRPC4 knockdown HRMECs cell culture, suggesting a role for TRPC4 as a modulator in retinal neovascularization (Song et al., 2015).

Furthermore, it was recently demonstrated that TRPC5 inhibition induces axon elongation through the optic nerve and, analogously, increases TRPC5 expression and decreases axonal extension, suggesting that TRPC5 modulates the projection of cell axonal processes of ganglion cells during and after retinal development (Oda et al., 2020).

TRPC1, C4, and C7 are expressed in the RPE, as represented in Figure 2D. Intracellular Ca^2+^ currents decreased considerably with specific TRPC subfamily blockers, suggesting that the basal Ca^2+^ influx, essential for RPE functions, is mainly due to the activity of TRPC (Wimmers and Strauss, 2007; Cordeiro and Strauss, 2011).

TRPM1 has been described in the INL, specifically in ON bipolar cells, as well as in the OPL of mouse, macaque, and human retina (Choi et al., 2015; Klooster et al., 2011; Križaj et al., 2010; Morgans et al., 2009; Figure 2D). It is well documented that ON bipolar cells hyperpolarize in response to glutamate, released in the dark, due the activation of mGluR6. The molecular mechanism involves the closure of TRPM1 through alpha and the beta-gamma complex of the Go-protein stimulated by mGluR6 (Koike et al., 2010; Xu et al., 2016). Thus, ON-bipolar cells show a depolarizing response to light, with the decrease of glutamate release. In agreement, TRPM1 knockout in mice results in a clinical pattern similar to the night blindness pathology (Morgans et al., 2009; Irie and Furukawa, 2014).

Previous studies have described how Mg^2+^ modulates some members of the TRP family (Huang et al., 2020; Sawamura et al., 2017; Zierler et al., 2017). Intracellular Mg^2+^ (1.7–2 mM) decreases the TRPM1 current in rod and ON-cone bipolar cells, and stimulation of the PLC-PKCα pathway alleviates this inhibition only in rod bipolar cells (Rampino and Nawy, 2011).

Recently, studies addressed the effects of TRPM2-mediated calcium influx when the channel was stimulated by oxidative stress, as well as the neuroprotective effect of antioxidants for TRP modulation in ARPE-19 cells and a role on sirtuin 2-dependent cell death (Figure 2D; Meléndez García et al., 2015; Daldal and Nazıroğlu, 2022a, 2022b).

In mice, TRPM3 has been found in the iris, ciliary body, retinal pigment epithelium, GCL, INL, IPL, and OPL (Brown et al., 2015; Hughes et al., 2012; Webster et al., 2020; Figure 2D). Expression of TRPM3 has also been described in Müller glial cells during mouse retinal development (Webster et al., 2020). A recent study showed an increase in Ca^2+^ influx induced by TRPM3 agonist into TRPM3-positive ganglion cells in developing mice, whereas TRPM3 knockouts presented a reduction in these currents (Webster et al., 2020). Knockouts for TRPM3, but not for TRPM1, have shown a lower percentage of pupil contraction in response to low light intensity stimuli when compared to control mice (Hughes et al., 2012).

TRPM4 is detected in the Müller glial, GCL, ONL, and, specifically, in cone photoreceptors of the human retina (Berdugo et al., 2021; Figure 2D). In addition, TRPM4 in the outer limiting membrane of the human retina partially colocalizes with internal rectifier potassium channel 6.2 (Kir6.2) and sulfonylurea receptor (SUR1), both subunits being constituents of ATP-dependent potassium channels (Berdugo et al., 2021; Omri et al., 2010). SUR1 or Kir6.2 also co-localizes with Müller glial cells and cone photoreceptors in monkey retinas (Berdugo et al., 2021).

RPE undergoes blue wavelength-induced apoptotic cell death. Blue light is emitted by sunlight and light-emitting diode (LED) lamps (Hu and Xu, 2021; Tao et al., 2019). TRPM7 overexpression protects RPE cells from cell death induced by blue light, while TRPM7 ablation results in a low proliferation rate and lower cell viability, a decrease in the levels of PKC, ERK, and Bcl-2 proteins, and an increase in Bax (Hu and Xu, 2021). These data suggest a key role for TRPM7 in the survival of RPE cells exposed to blue light via PKC/ERK signaling (Hu and Xu, 2021).

TRPP2 has been detected in all layers of the mouse retina, with strong intensity in the outer segments of cones and INL (Gilliam and Wensel, 2011; Figure 2E). Patients with ML-IV have severe deleterious neurological, motor, and ocular consequences (Smith et al., 2002; Grishchuk et al., 2016). In the genetic ablation of TRPML1, it was seen that there was a thinning of the ONL and shortening of the outer segments of the rods in an age-dependent manner. Furthermore, except for INL, accumulation of lysosomal aggregates was found in almost all retina layers in *Trpml1^−/−^* (Grishchuk et al., 2016).

In animals with retinal detachment, agonist stimulation of TRPML1 decreases rod and cone apoptosis, preserving the photoreceptor layer. In addition, the activity of the channel changes in these retinas and therefore, ROS concentrations are markedly smaller. In this way, there is an improvement in the visual capacity in the injured animals treated with an agonist. These results make the channel an excellent therapeutic target (Yan et al., 2021).

Except for TRPML, TRPP1-3 mRNA expression has been demonstrated in the optic nerve. Furthermore, TRPP1-2 is one of the most abundant in astrocytes around the ocular structure (Choi et al., 2015). Mucolipidosis IV can also induce optic nerve thinning and neuronal demyelination at the level of the brain, which results in severe motor problems (Smith et al., 2002; Grishchuk et al., 2016). As expected, TRPML1 knockout mice show a decrease in the thickness of myelinated axonal projections and optic nerve thickness, possibly caused by a decrease in blood supply (Grishchuk et al., 2016).

Toll-like receptor 3 (TLR3) has recently been linked to ATP production from lysosomes in RPE cells and astrocytes expressed in the murine optic nerve head (ONH). The activation of TLR3 in these cells induces the stimulation of TRPML1, which, in turn, by inducing the release of lysosomal Ca^2+^ into the extracellular environment, can promote the fusion of the plasma membrane with the lysosomal membrane and increase the release of ATP from the organelle (Beckel et al., 2018). Gomez and colleagues (2018) observed, in *Trpml1^−/−^* mice, low extracellular Ca^2+^ concentrations even with pharmacological stimulation and confirmed that ion efflux occurred through channel activity (Gómez et al., 2018).

It is known that the inadequate functioning of the lysosome can lead to the accumulation of metabolites, including lysosomal lipids, which generate oxidative stress in the tissue. Experimental data indicate that TRPML1 gene expression remained at normal levels in RPE, but extracellular Ca^2+^ elevation was impaired under conditions of lipid accumulation, suggesting that TRPML1 responses are essential for lysosomal ATP release (Gómez et al., 2018).

Finally, TRPVs are expressed in astrocytes, microglia, and endothelial cells (Ho et al., 2014; Leonelli et al., 2010). TRPV1 is located in all retina layers during development and in adult mice. V1 shows higher immunofluorescence intensity in INL, GCL, and IPL in the mature mouse retina, while V2 is weakly restricted to INL, OPL, IPL, and GCL (Choi et al., 2015; Leonelli et al., 2010; Leonelli et al., 2009). As illustrated in Figure 2C, TRPV1 is expressed in amacrine cells, and TRPV4 was recently found in bipolar cells, while the V4 homodimer was found in Müller glia and RCGs. However, there are also reports of TRPV1/TRPV4 co-localization in these regions of the retina of mice (Gao et al., 2019; Leonelli et al., 2011; Sappington et al., 2015).

In the retina, TRPVs play a role in mechanical damage (Ho et al., 2014; Leonelli et al., 2010). As reported by Ho et al. (2014), in astrocytes that express TRPV1 and when scratching retinal tissue, there is an increase in the shortening and migration of these cells, through an increase of intracellular Ca^2+^ mediated by the channel in response to mechanical stress (Ho et al., 2014).

Interestingly, TRPV1 levels increase in the GCL and optic nerve axons after optic nerve injury in mice, whereas 21 days after injury, there is a decrease in TRPV1 staining in microglia and astrocytes. On the other hand, capsaicin, a TRPV1 agonist, accelerated cellular degeneration, induced retinal thinning, and intensified glial fibrillary acidic protein (GFAP) immunofluorescence in Müller’s glia. Capsaicin increases the generation of lipid peroxidation products *in vitro* but not *in vivo*, and this response is blocked by receptor antagonist capsazepine (Leonelli et al., 2010).

TRPV1-4 is expressed in the RPE of mice and is a target of type 1 angiotensin II receptor (AT1R) signaling triggered by its activation by angiotensin II (AngII) (Barro-Soria et al., 2012). Indirect activation of V2 contributes to Ca^2+^ influx to the cytoplasm and release of ions stored in the endoplasmic reticulum. This role, attributed to TRPV2 in RPE was observed through the inhibition of AngII, caused by the application of the PLC blocker, abolishing the influx of Ca^2+^ and demonstrating the involvement of the pathway. Corroborating with the data reported above, the researchers also used SKF96365, a TRPV2 antagonist, and AngII simultaneously to evaluate the channel activation responses by AngII. As expected, the Ca^2+^ currents were suppressed in RPE cultures (Barro-Soria et al., 2012).

Similarly, VEGF production is also mediated by the interaction of the insulin-like growth factor (IGF)-1 receptor with AngII, which stimulates phosphoinositide 3-kinase (PI3K) activity to induce Ca^2+^ influx into TRPV1 and V2. Interestingly, IGF-1 increases the expression of TRPV2 channels, and inhibition of IGF-1 and its specific receptor leads to a drastic reduction in the effects of the Ang II pathway and in the formation of new blood vessels in the human RPE in a pathological context, thus suggesting a relationship between TRPVs and neovascularization (Cordeiro et al., 2010; Reichhart et al., 2015; Smith et al., 1999).

The optic nerves of mice and rats have been found to express TRPA1, TRPM1-M4, M6-M7, TRPC1-C6, TRPV1-V2, and V4 (Choi et al., 2015; Leonelli et al., 2009; Papanikolaou et al., 2017).

## 5 Retinal pathologies

### 5.1 TRPs, angiogenesis, and age-related macular degeneration

Age-related macular degeneration is a leading cause of blindness, affecting 200 million people around the globe. With expectations to increase in the next few years, AMD represents a major public health problem (Wong et al., 2014). It is a multifactorial disorder that is driven by genetic and environmental influences. The disease affects the macular region of the retina, a cone-enriched region of high-acuity vision, particularly in the primates. Pathogenesis includes characteristic lesions through the formation of drusen or basal laminar deposits at initial stages, while neovascular abnormalities account for late-stage phenomena in the wet or exudative AMD, but not for dry AMD (Mitchell et al., 2018).

Genome-wide association studies have been performed throughout the years to identify candidate genes associated with the progression of AMD (Fritsche et al., 2014). Recently, two studies identified the TRPM1 gene as one of the candidates correlated to AMD incidence (Persad et al., 2017; Orozco et al., 2023). Although there is little evidence of a direct correlation between TRP channels and the progression of AMD, there are associations that could be made to provide new insight into this area (Wong and Yao, 2011).

Wet AMD is a treatable form of the disease, and anti-VEGF compounds represent the current treatment, since VEGF is one of the most studied angiogenic factors leading to neovascularization. Endothelial cells (EC) express many forms of TRP family members (Wong and Yao, 2011) and are key cell types to promote angiogenesis. Angiogenic factors like VEGF can activate TRP channels, increasing intracellular Ca^2+^ and modulating angiogenic pathways in a diversity of situations (Kwan et al., 2006; Smani et al., 2018; Negri et al., 2019).

Several studies demonstrate the coupling of TRP channels to angiogenesis in the retina, which is essential to understanding the potential of these proteins to modulate neovascular disease progression, like AMD. It has been shown that the silencing of TRPC4 with siRNA inhibits neovascularization in an oxygen-induced retinopathy (OIR) model performed from P7 to P12 mice, and evaluated at P17 when neovascularization peaks (Song et al., 2015). This model is important to simulate and understand conditions like retinopathy of prematurity, a leading cause of blindness among children. These results were replicated in HRMECs in which silencing TRPC4 inhibited VEGF-induced migration and tube formation capacity, also inhibiting signaling pathways triggered by the vascular growth factor, indicating that this channel is crucial for VEGF effects (Song et al., 2015).

Using the same parameter of OIR, Zhu et al. (2019) found that TRPC5 is also a regulator of angiogenesis in mouse retina, seeing that TRPC5 knockout reduces sprouting and tube formation and increases avascular areas and neovascular tufts. The mechanism of TRPC5 signaling seems to involve the activation of nuclear factors activating T cell isoform c3 (NFATc3) and angiopoietin-1 (Zhu et al., 2019). The study also suggests that the FDA approved drug, riluzole, used for amyotrophic lateral sclerosis (ALS) and activator of TRPC5, could be an option to treat ischemic diseases, as it increased perfusion in the hindlimb ischemic model.

**Figure 3:**
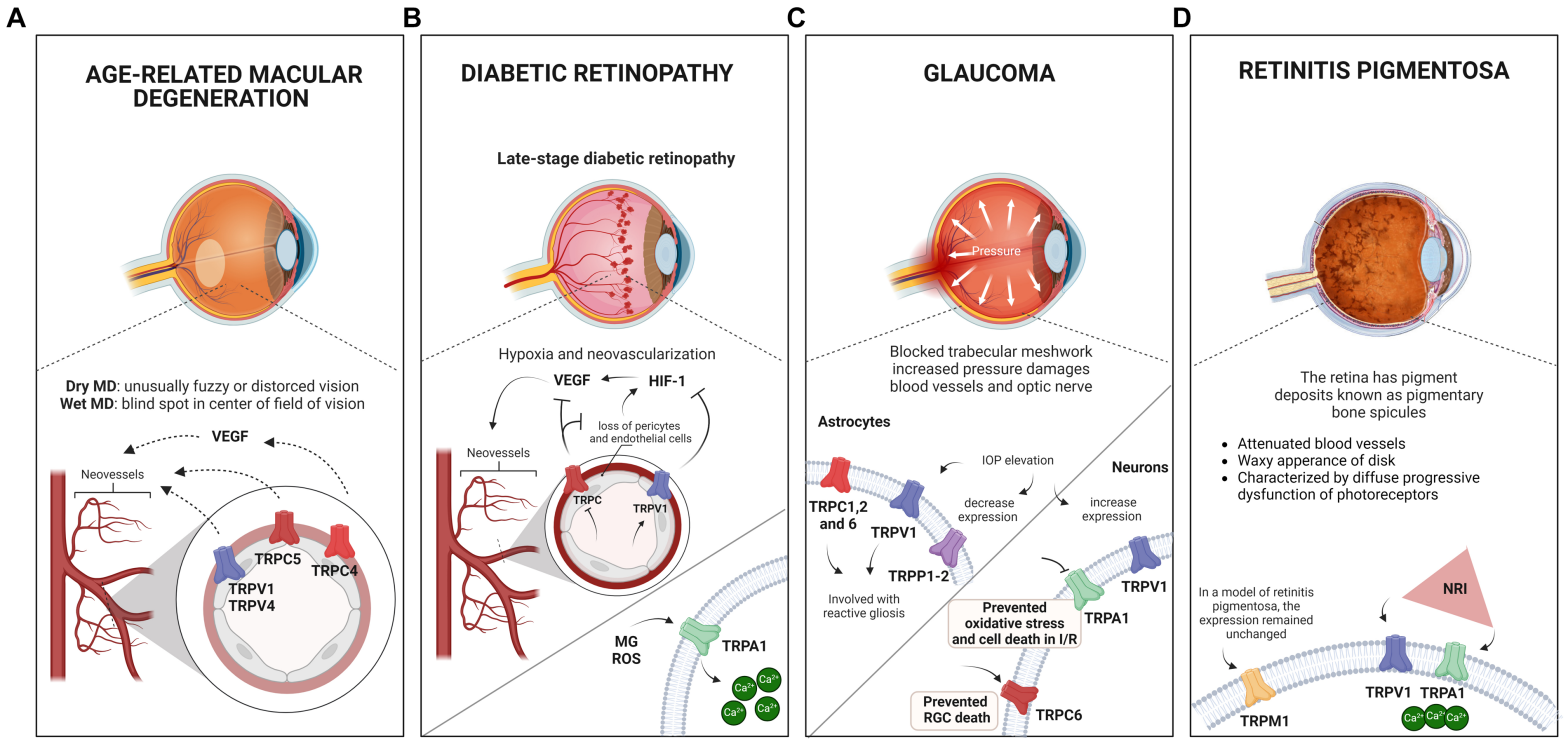
Eye pathologies and the involvement of TRP channels. **(A)** Schematic illustration of macular degeneration. In the wet form of the disease, abnormal blood vessel growth in the retina can lead to fluid and blood leakage, damaging the macula and causing sudden or rapid loss of central vision. Research has suggested that when activated, TRPV1, TRPV4, TRPC5, and TRPC4 receptors may contribute to the neovascularization process associated with the disease. Notably, TRPC4 activation occurs via VEGF stimulation. **(B)** Schematic showing the late stage of diabetic retinopathy (DR). Disruption of the blood-retinal barrier results in microaneurysms, increased vascular permeability, and capillary obstruction, leading to inadequate retinal oxygen distribution. Increased VEGF expression and hypoxia-inducible factor 1 (HIF-1) activity have been observed, suggesting the concept of DR to neurovascular diseases. Studies have indicated that TRPV1 activation suppresses the PPARγ-poldip2-Nox4 pathway, thereby inhibiting DR-associated increases in HIF-1, VEGF, ROS, and H₂O₂, while reducing retinal microvessel hyperpermeability. In addition, TRPC inhibition decreased VEGF expression and inhibited neovascularization. TRPA1 activation by methylglyoxal (MG) and reactive oxygen species (ROS) increases Ca^2+^ levels, that could lead to tissue damage and exacerbation of oxidative stress. **(C)** Schematic illustrating glaucoma and its association with elevated intraocular pressure (IOP). Glaucoma complex mechanisms, including genetic mutations, reduced ocular blood supply, neurotoxicity due to increased ROS production, cell death, and phagocytosis. In astrocytes, TRPCs, mainly TRPC1, TRPC2, and C6, appear to contribute to the disease, particularly to reactive gliosis. This can also be induced by elevated IOP, which activates TRPV1 receptors. Studies have revealed a significant decrease in TRPP1-2 expression 24 h after IOP elevation. In neuronal cells, elevated IOP increases TRPV1 expression in the inner plexiform layer (IPL) and retinal ganglion cells (RGCs), leading to an elevated rate of spontaneous action potential. The protective role of TRPV1 in ganglion cells promotes the stress response, and the absence of the channel appears to accelerate degeneration. Inhibition of TRPA1 during ischemia protects against retinal cell death in chick and mouse models, preventing oxidative stress and cell death induced by IOP reperfusion, highlighting its role in triggering the degeneration and elevation of IOP in glaucoma. Studies have also suggested that TRPC6 may mediate a neuroprotective response in a model of ischemia and reperfusion by increasing the number of ganglion cells. **(D)** Schematic illustrating retinitis pigmentosa, characterized by pigment deposits known as pigmentary bone spicules. TRPM1 expression in Rd1 mice (a model of retinitis pigmentosa) remained similar to that in wild-type mice at P21, despite retinal remodeling due to degeneration. Near-infrared (NIR) light therapy has been shown to protect the retina against neurodegeneration and inflammatory processes in a retinitis pigmentosa model. This treatment stimulates retinal cell signaling without causing tissue damage, increases Ca^2+^ influx, and triggers ganglion cell responses. The thermal sensitivity of TRPV1 and TRPA1 channels to the treatment was evaluated based on their sensitivity to temperature variations.

The role of TRPV1 and 4 was also investigated in HRMECs. Pharmacological inhibitors of TRPV1 or 4 channels reduce sprouting angiogenesis, although they are ineffective in reducing VEGF-induced sprouting, suggesting that these channels are not required for the vascular factor’s effect (O'Leary et al., 2019). Interestingly, inhibition of the channels reduces neovascularization while increasing normal vascular area in the OIR model. Both channels have been linked to involvement in corneal injury (Okada et al., 2021); thus, *in vivo* and *in vitro*, genetic or pharmacological ablation reduced fibrotic proliferation and inflammatory cytokines in the case of V4 (Okada et al., 2016), in addition to the absence of V1 impairing wound healing and cell migration (Sumioka et al., 2014). Therefore, modulation of TRP channels could arise as alternative treatments for patients not responding to regular therapeutic methods in ischemic and neovascular retinal diseases. Figure 3A illustrates the putative relationship between the activity of TRP channels and AMD.

### 5.2 Diabetic retinopathy

Diabetic retinopathy (DR) is among the leading causes of blindness in working-age adults. Classically and clinically, DR has been considered a vascular disease (Wang and Lo, 2018; Stitt et al., 2016). A key event is the blood-retinal barrier disruption, with loss of pericytes and endothelial cells, leading to microaneurysms, increased vascular permeability, and capillary obstruction, resulting in poor oxygen distribution in the retina (Wang and Lo, 2018; Sachdeva et al., 2018; Stitt et al., 2016). Consequently, VEGF has increased expression through the hypoxia-inducible factor 1 (HIF-1) (Wang and Lo, 2018). However, a great amount of data has demonstrated many early modifications in neural aspects of retinal physiology, extending the current concept of DR to a neurovascular disease (Carpi-Santos et al., 2022). Blindness arising from this pathology can result from different mechanisms. The most frequent is due to diabetic maculopathy, characterized by an increased concentration of macular fluid (Wang and Lo, 2018; Stitt et al., 2016).

The role of oxidative stress in the retina of hyperglycemic patients is well documented, being marked by accentuated production of ROS, as well as RCS and RNS (Wang and Lo, 2018; Sachdeva et al., 2018). Additionally, a sustained generation of MG promotes a series of structural changes in macromolecules and transcriptional modifications (Sachdeva et al., 2018; Schlotterer et al., 2019), and other end products of advanced glycation (AGEs) are generated from MG (Sachdeva et al., 2018; Schlotterer et al., 2019; Stitt et al., 2016).

TRPA1 is activated by MG (Andersson et al., 2013; Eberhardt et al., 2012) and ROS (Andersson et al., 2008; Mori et al., 2016), through covalent modifications of N terminal cysteine residues (Hinman et al., 2006), increasing Ca^2+^ levels, leading to tissue damage and exacerbation of oxidative stress, as shown in Figure 3B. There is evidence that hypoxia can lead to TRPA1 activation through produced-lipid peroxidation metabolites (Pires and Earley, 2018). Conversely, TRPA1 knockout or pharmacological inhibition plays a pivotal role in reducing ischemia/reperfusion oxidative damage and apoptosis in the retina (de Araújo et al., 2020), in traumatic brain injury (Yang et al., 2022), and preventing itching and hyperalgesia in mice with methylglyoxal and streptozotocin-induced diabetes, with similar effects to methylglyoxal scavengers, such as D-arginine and aminoguanidine (Cheng et al., 2019). TRPA1 deficiency and antagonism are also able to reduce hypoxia-induced painful dysesthesia, similarly to ROS scavengers (So et al., 2016), and attenuate cold hypersensitivity caused by diabetic peripheral neuropathy (Hiyama et al., 2018). Since DR progression depends on oxidative stress (Carpi-Santos et al., 2022), these results raise an interesting possibility of searching for the role of TRPA1 in DR models. An exciting target to study TRPA1 is Müller glia, which express TRPA1 (de Araújo et al., 2020), and show oxidative stress as an early response to hyperglycemia conditions, upregulating VEGF (Rossino et al., 2020), which contributes to the injurious events in DR, such as increased endothelial permeability, angiogenesis, and apoptosis (Wang et al., 2015). As oxidative stress is a key stimulus to several biochemical and neurochemical alterations in DR (Fanaro et al., 2023), and VEGF is a crucial signal to induce angiogenesis, the study of TRPA1 in Müller glial cells appears to be a promising target to develop future therapeutic agents.

Several studies also point to a strong impact of TRPV channels in DR pathogenesis. For instance, TRPV1 activation by capsaicin suppresses the PPARγ-poldip2-Nox4 pathway, inhibiting DR increases in HIF-1, VEGF, ROS, and H₂O_2_ and decreased hyperpermeability of retinal microvessels (Liu et al., 2022). Moreover, TRPV4 is downregulated by hyperglycemia in diabetic rats, which suggests a possible contribution to protection from endothelial dysfunction in diabetes (Monaghan et al., 2015). In one study, this channel’s inhibition by vasoinhibins prevented excessive blood retinal barrier (BRB) permeability in diabetes (Arredondo Zamarripa et al., 2017). Another study found that TRPV4 gene deletion abolished retinal edema, which is highly important to BRB disruption, corroborating the possible role of BRB dysfunction in DR (Orduña Ríos et al., 2019). Regarding neuronal dysfunction, it has already been described that TRPV4 contributes to ganglion cell stretch and to the activation of proapoptotic signaling pathways in the mouse retina (Ryskamp et al., 2011), which could, therefore, contribute to an increase of damage in DR. Therefore, modulation of TRPV4 in early periods of DR, which shows harmful effects on neural retina, with no angiogenesis effect, could be harmful to the progression of DR. The data suggest that TRPV4 could be an attractive target to study in the context of later stages of DR with neovascularization.

TRPC channels are widely present in the retina and have been studied as possible targets for their contribution to exacerbating DR harm. Although their expression in hyperglycemic contexts needs to be better established, there has been evidence of an increase in a diabetic model compared to control (Sachdeva et al., 2018). STZ-treated *TRPC1/4/5/6^−/−^* mice presented decreased endothelial and pericyte cell death that led to retinal thickness preservation, as well as lower concentrations of MG in plasma and red blood cells, and enhanced expression and activity of glyoxalase 1 (GLO1), the major MG detoxification enzyme, compared to control (Sachdeva et al., 2018). TRPC channels have also been strongly associated with angiogenesis in several models, mediating VEGF-induced Ca^2+^ influx (Smani et al., 2018). Moreover, TRPC inhibition by SKF96365 decreased VEGF expression and downregulated proliferation, migration, and lumen formation in HRECs exposed to high glucose, inhibiting neovascularization (Lang et al., 2020). Another study documented aggravation in the pathogenesis of DR by activating TRPC6 by Hyp9, a highly selective TRPC6 agonist, increasing ROS accumulation and enhancing the augmented expression of IL-6 and VEGF in cells exposed to high glucose. Adversely, its silencing and knockdown inhibited the decrease in Müller cell viability and gliosis in rMC-1 cells while decreasing IL-6, VEGF, ROS, and intracellular Ca^2+^ levels.

TRPM2 was shown to be activated by hypoxia-induced ADP-ribose and oxidative stress, common events in DR pathogenesis, and its blockage restored pro-inflammatory cytokines (TNF-α and IL-1β) and ROS levels (Özkaya et al., 2021). In contrast, TRPM8 activation by linalool produces anti-angiogenic effects through inhibition of beta 1 integrin/focal adhesion kinase (FAK) signaling (Becker et al., 2021), which could be of great relevance to attenuating DR implications.

Together, these findings portray the relevance of TRP channels in diabetic retinopathy pathogenesis, each type presenting its characteristics in the signaling pathways. It is important to highlight, however, that information about TRP channels in DR is still very scarce, and further research is needed. Considering the effects of some TRPs in the retinal function, together with the scarce data available in the DR context, it would be interesting to invest in the better characterization of the potential protective effect of the inhibition of TRPV1, TRPV4, TRPC1/4/5/6 in later stages of DR, due to their regulation of angiogenesis. On the other hand, the inhibition of TRPA1 and TRPM2 could be interesting in the context of early alterations that occur in the neural retina of diabetic animals. Finally, the activation of TRPM8 could also be a promising target to evaluate a protective effect in the DR context with neovascularization.

### 5.3 Glaucoma

Glaucoma is a group of ophthalmological conditions that leads to retinal/optic nerve degeneration, initially affecting the periphery of the retina (Fan Gaskin et al., 2021; Guedes, 2021; Jayaram et al., 2015). It is estimated that about 76 million individuals are affected by glaucoma, representing the main cause of irreversible vision loss.

Depending on the causes that initiate and develop the disease, it is possible to divide glaucoma into primary and secondary (Moazzeni et al., 2020). Although open-angle glaucoma is the most common type of neuropathy, representing around 80% of cases (Guedes, 2021; Moazzeni et al., 2020; Okumus et al., 2013), both primary open and closed-angle glaucoma involve a complex and heterogeneous set of mechanisms, such as increased intraocular pressure (IOP), genetic mutation, decreased blood supply to the eye, neurotoxicity from increased ROS production, cell death, and phagocytosis (Fan Gaskin et al., 2021; Moazzeni et al., 2020; Okumus et al., 2013). Thus, IOP regulation is currently the main approach for clinical treatment, intervention, and subject of study aiming for a better understanding of glaucoma (Jayaram et al., 2015). Despite data implicating TRP family channels in ocular pathologies (Choi et al., 2015; Irnaten et al., 2020; Oda et al., 2020), research on the role of TRP in glaucoma is still scarce.

The role of TRPA1 in retinal ischemic conditions has been evaluated, and the inhibition results to be a protective effect. Treatment with a first generation TRPA1 antagonist (HC-030031) during acute ischemia promotes a protection of cell death in the chick retina (Araújo et al., 2017). In a murine model of glaucoma, pharmacological inhibition (A-967079 or HC-030031) or genetic ablation of TRPA1 also prevents oxidative stress and cell death promoted by IOP-reperfusion (2 and 7 days) in mouse retinas (de Araújo et al., 2020). These findings point to an active role of TRPA1 in modulating degeneration in ischemic conditions and IOP present in glaucoma regulating oxidative stress (de Araújo et al., 2020; Araújo et al., 2017; Ling et al., 2016). These data reveal a promising possibility for developing new treatments and encourage research to describe better the protective mechanisms involved. In addition to the retina, TRPA1 is also present in intraocular trigeminal nerves (TG) (Ling et al., 2016). Since previous data state that the channel responds to membrane stretching and is expressed in TG, it could be involved with regulating intraocular pressure. Accordingly, Bimatoprost, a prostanoid analog used to control IOP in glaucomatous patients, can activate TRPA1 in TG neurons (Ling et al., 2016). Thus, it would be interesting to test the effect of Bimatoprost on retinal cell death detected in different glaucoma models.

TRPCs, mainly TRPC1 and TRPC2 and C6, have been detected in the optic nerve and adjacent astrocytes and appear to play a role in this eye disease, as shown in Figure 3C (Choi et al., 2015; Molnár et al., 2016; Irnaten et al., 2020). In mice with glaucoma, *Trpc1^−/−^* exhibited marked and rapid reactive gliosis when compared to the wild type; the authors suggest that this is due to the mechanosensitive properties of TRPC1 in response to ocular pressure, which would be involved in the suppression of gliosis (Molnár et al., 2016).

The increase in IOP also causes significant changes in the lamina cribrosa (LC), which is responsible for involving the optic nerve. This region is obstructed, making it difficult for axons to exit the retina to the brain nuclei. In addition, LC is rich in astrocytes that organize the exit of axons from the eye, and these astrocytic cells become reactive in the face of some damage, such as optic nerve crush. Another important finding in LC in glaucomatous is that there is an increase in matrix proteins that induce the development of fibrous tissue in response to oxidative stress in the tissue. Thus, it has been reported that both gene silencing and pharmacological inhibition of TRPC1 and C6 reduce matrix protein gene expression, suggesting that TRPC channels play a role in the oxidative context involving LC with glaucoma (Irnaten et al., 2020). On the other hand, an increase in intracellular Ca^2+^ in LC has been reported, possibly due to TRPC activity (Irnaten et al., 2020). It has been suggested that the influx of extracellular Ca^2+^ also acts on the regulation of the contractile properties of trabecular meshwork (TM) via TRPC1 and TRPC4 (Abad et al., 2008), possibly via depletion of intracellular Ca^2+^ stock concentrations in the endoplasmic reticulum, a mechanism similar to that described for the activation of SOCE channels (Abad et al., 2008; Yuan et al., 2009). In addition, it has been shown that TRPC5 modulation regulates axon growth in the optic nerve, with TRPC5 inhibition promoting the elongation of axons while its activation reduces axon extension (Oda et al., 2020). Interestingly, in ganglion cells, there is a temporary increase in TRPC6 mRNA in response to ischemia and reperfusion in mice. In addition, the stimulation of TRPC6 with OAG in injured retinas increases the number of RGCs, whereas its inhibition triggers a decrease in ganglion cells. These data suggest that TRPC6 may mediate a neuroprotective response in an ischemia and reperfusion model (Wang et al., 2010). Therefore, in the context of glaucoma, which shows ganglion cell axon degeneration in the optic nerve, this seems to be a good opportunity to study the protective effect of the inhibition of TRPC5 together with the activation of TRPC6. It would also be interesting to evaluate the possible facilitation by the combination with inhibitors of TRPC1 to assess the elongation of the ganglion cell axons due to the reduction of matrix proteins and the development of fibrous tissue in LC.

In wild-type mice, expression of TRPP1-3, TRPP1 is much lower than TRPP2, while TRPP3 is weakly marked in ONH. In addition, elevated levels of TRPP1-2 have also been found in ONH-associated astrocytes. However, after 24 h of IOP elevation, there was a marked decrease in TRPP1-2 expression (Choi et al., 2015). Although there are very few studies evaluating TRPPs in glaucoma, the presence and the alteration observed in IOP indicate that they could represent an interesting target for the study of the axon degeneration present in this retinopathy.

IOP elevation increases TRPV1 expression in IPL and RGCs, accompanied by an increase in spontaneous action potential rate, and in *Trpv1^−/−^* ganglion cells, the high firing rate was not observed (Weitlauf et al., 2014). This suggests a protective role of TRPV1 in ganglion cells by promoting a stress response and, furthermore, the absence of the channel appears to accelerate degeneration (Weitlauf et al., 2014).

On the other hand, Sappington et al. (2015) reported that both TRPV1 knockout and pharmacological inhibition of the channel in an *ex vivo* model of increased IOP, resulted in fewer RGCs labeled for an apoptotic marker, suggesting that the entry pathway of TRPV1 Ca^2+^ could be related to cell death (Sappington et al., 2015). Corroborating these data, McGrady and colleagues observed no changes in pressure in the presence or absence of TRPV1 (McGrady et al., 2020; Risner et al., 2020), and TRPV1 knockouts did not show any protection against IOP-induced cell death after 2 or 7 days of reperfusion (de Araújo et al., 2020).

Intraocular injection of capsaicin promotes microglial gliosis (Leonelli et al., 2010). Interestingly, in an animal model, increased IOP leads to Ca^2+^ influx via TRPV1, which, in turn, stimulates interleukin-6 (IL-6) production and secretion by retinal microglia close to ganglion cells. However, in cell culture, pharmacological inhibition of TRPV1 associated with increased hydrostatic pressure resulted in decreased cytokine levels (Sappington and Calkins, 2008).

TRPV1 retinal content is inversely regulated by IOP in young and old mice, with lower pressure increasing TRPV1 expression (and *vice-versa*) (Sappington et al., 2015), as shown in Figure 3C. It appears that RGC also responds to an increase in salinity with Ca^2+^ influx through TRPV4, and prolonged periods in this condition decrease the viability of these cells (Ryskamp et al., 2011).

Other studies have demonstrated the presence of TRPs in different structures involved in the pathophysiology of glaucoma and raised the possibility of their involvement. In the case of TRPM3, it is possible that the increase in Ca^2+^ influx, mediated by the channel, participates in trabecular contraction and relaxation to promote contraction of the smooth muscle associated with these regions (Bennett et al., 2014; Hughes et al., 2012; Wiederholt et al., 2000).

It is well documented that these contractions of the trabecular meshwork coordinate the inflow and outflow of aqueous humor (Wiederholt et al., 2000), and that the ciliary body also plays a role in fluid production (Bennett et al., 2014). Thus, the balance between the production and drainage of aqueous humor is directly associated with IOP stability. Furthermore, as TRPM3 is abundantly expressed in both the trabecular meshwork and ciliary body, it is possible that the channel has a role in maintaining IOP by promoting a sustained Ca^2+^ current (Bennett et al., 2014; Shiels, 2020).

The TRPM3 gene is composed of an intron that harbors the non-coding micro-RNA (miR204) (Jayaram et al., 2015; Shiels, 2020). miR204 is one of the most abundant micro-RNAs in most layers of the human retina, excluding only IPL and OPL. In addition, the micro-RNA is also one of the most expressed in retinal regions that suffer ocular damage, but in a murine model of glaucoma, miR204 is downregulated, possibly via TGF-β modulation (Jayaram et al., 2015; Bennett et al., 2014). In glaucoma, several studies have reported that increased TGF-β concentration is associated with the emergence of dense fibers in the TM, contributing to increased IOP (Jayaram et al., 2015; Robertson et al., 2010; Shiels, 2020).

### 5.4 Retinitis pigmentosa

Retinitis pigmentosa (RP) comprises a group of neurodegenerative and genetic diseases, the heredity causes for which may be autosomal recessive, dominant, X-linked, or mitochondrial (Niwa et al., 2016). Furthermore, the pathology may result in visual loss alone and be classified as “non-syndromic” RP or, if associated with other syndromes such as Usher and Bardet-Biedl syndrome, be categorized as “syndromic” RP (Daiger et al., 2014; O'Neal and Luther, 2024). The visual deficit arises from the degeneration of photoreceptors, first rods, followed by cones (Guadagni et al., 2015).

In ophthalmological examinations of the fundus of the eye, the depression of blood vessels can be observed; bone spicules between photoreceptors that morphologically alter the environment and facilitate the diffusion of non-resident cells (Daiger et al., 2014).

There is still no cure for the disease, given the variety of genetic mutations responsible for the appearance of retinitis pigmentosa; however, one alternative is the development of palliative treatments aimed at prolonging the useful life of photoreceptors, delaying degeneration (Hartong et al., 2006; Massengill et al., 2020). Recently, Nelidova and colleagues studied near-infrared (NIR) light therapy in the Rd1 model involving TRPV1 and TRPA1 channels (Nelidova et al., 2020). Since both thermal activity and the presence of these channels are well documented in the vertebrate retina, the group used a gold nanomaterial viral system. Thus, by exciting the nanorods with NIR, heat is generated; this thermal energy activates TRPV1 and TRPA1 expressed in the surviving functional cones, and the response extends to the GCL of the Rd1 retina, a result similar to that obtained in the wild-type (Nelidova et al., 2020). This type of treatment is based on the concept that light absorption in the low radiation range could stimulate and modulate retinal cell signaling without causing tissue damage. When cones are stimulated with NIR, Ca^2+^ influx increases and triggers ganglion cell responses (Zhu et al., 2021). NIR also increases the activity of neurons in the visual cortex in animal models of retinitis pigmentosa when compared to control (Nelidova et al., 2020). Figure 3D summarizes the present data of TRAP1 and TRPV1 in RP.

Finally, the expression of TRPM1 in Rd1 mice, one of the animal models for retinitis pigmentosa, was similar to the wild type at P21 despite retinal remodeling occurring due to degeneration (Križaj et al., 2010).

The few data available on TRPs in retinitis pigmentosa highlight the need to explore this field for an innovative and effective treatment. In addition, we currently do not find any data to address the signaling mechanisms that trigger the symptoms described involving any TRP (Maneu et al., 2022; Nelidova et al., 2020; Zhu et al., 2021).

## 6 Concluding remarks

TRPs are a heterogeneous family of ion channels with some structural similarities, highly modulated by endogenous and exogenous stimuli. It has been demonstrated that potentially harmful sensory and mechanical stimuli can modulate TRPs in physiological and pathological contexts. Indeed, TRPs are important for development and maintenance health and retinal pathologies.

The present review discusses the potential role of TRP channels in the pathophysiology of, and possible therapeutic targets for, the most prevalent retinal diseases. Several studies have revealed that the TRP channel family is ubiquitously present in the retina; these studies shed light on findings demonstrating their involvement in the physiology of virtually all retinal cells. Using different disease models, much evidence shows their clear involvement in distinct retinopathies. Here, we highlighted the studies that analyze strategies to delay or prevent the progression of these diseases using TRP modulation, with some encouraging results, as well as highlighting unanswered questions yet to be studied.

It is extremely important to understand the factors, agents, and biochemical and/or genetic contexts that induce the appearance of the pathologies addressed in this review. It is also necessary to have therapeutic approaches that promote neuroprotection, alleviating degeneration with no or few side effects, through, for example, drugs that modulate TRP channels.
