# Supplementary material for: Functions of TRPs in retinal tissue in physiological and pathological conditions
Source: Front Mol Neurosci. 2024 Sep 25;17:1459083. doi: 10.3389/fnmol.2024.1459083 (PMC11461470; doi:10.3389/fnmol.2024.1459083)
Supplement: Supplementary file 1 [file Table_1.docx]

Supplementary Material

Functions of TRPs in vertebrate retinal tissue in physiological and pathological conditions

**Nascimento, THO^1^; Pereira-Figueiredo, D^2^; Resende, LV^2^; Nascimento, AA^2^, DeLogu, F^5^ Nassini, R^5,^ Campello-Costa, P^3^; Melibeu-Faria, AC^4^; Araújo, DSM^5^; Calaza KC^1,2^**

*** Correspondence:** Corresponding Author: email@uni.edu

Supplementary: Scheme of the article search methodology for this review.

**“STRUCTURE”**

**“MODULATION”**

**TRPA1 = 62**

**TRPC = 46**

**TRPM = 38**

**TRPML = 11**

**TRPP =11**

**TRPV = 90**

**“SIGNALING”**

**TRPA1 = 95**

**TRPC = 105**

**TRPM = 74**

**TRPML = 27**

**TRPP = 47**

**TRPV = 121**

**TRPA1 = 31**

**TRPC = 33**

**TRPM = 42**

**TRPML = 11**

**TRPP = 17**

**TRPV = 63**

- TOTAL Nº OF ARTICLES WITH RESPECTIVE KEYWORDS:
- TOTAL Nº OF ARTICLES WITH RESPECTIVE KEYWORDS:

**TRPA1 = 11**

**TRPC = 11**

**TRPM = 12**

**TRPML = 6**

**TRPP = 6**

**TRPV = 14**

**“PIGMENT EPITHELIUM”**

**“OPTIC NERVE”**

**“RETINAL”**

**“RETINA”**

**TRPA1 = 2**

**TRPC = 0**

**TRPM = 2**

**TRPML = 2**

**TRPP = 0**

**TRPV = 0**

**TRPA1 = 2**

**TRPC = 2**

**TRPM = 1**

**TRPML = 0**

**TRPP = 0**

**TRPV = 1**

**TRPA1 = 8**

**TRPC = 9**

**TRPM = 13**

**TRPML = 8**

**TRPP = 4**

**TRPV = 13**

**TRPA1 = 1**

**TRPC = 1**

**TRPM = 0**

**TRPML = 0**

**TRPP = 0**

**TRPV = 0**

**TRPA1 = 0**

**TRPC = 0**

**TRPM = 0**

**TRPML = 0**

**TRPP = 0**

**TRPV = 1**

**TRPA1 = 6**

**TRPC = 0**

**TRPM = 0**

**TRPML = 0**

**TRPP = 0**

**TRPV = 1**

**TRPA1 = 3**

**TRPC = 13**

**TRPM = 4**

**TRPML = 1**

**TRPP = 4**

**TRPV = 41**

**“RD1”**

**“GLAUCOMA”**

**TRPA1 = 0**

**TRPC = 13**

**TRPM = 2**

**TRPML = 0**

**TRPP = 0**

**TRPV = 17**

**TRPA1 = 2**

**TRPC = 25**

**TRPM = 10**

**TRPML = 3**

**TRPP = 1**

**TRPV = 39**

**TRPA1 = 3**

**TRPC = 12**

**TRPM = 5**

**TRPML = 2**

**TRPP = 0**

**TRPV = 15**

**TRPA1 = 3**

**TRPC = 21**

**TRPM = 8**

**TRPML = 1**

**TRPP = 0**

**TRPV = 15**

**“MACULAR DEGENERATION”**

**“METHYLGLYOXALE”**

**“DIABETIC RETINOPATHY”**

**“INTRAOCULAR PRESSURE”**

**“RD10”**

**“RETINITIS PIGMENTOSA”**

Scheme 1: Representation of the search strategy for the studies included in the present paper. For all topics covered in the review, the selection of articles was made by combining the keywords, located in the longitudinal box on the left, with the name of each channel in the family, located in the box on the right. The total number of articles found corresponds to the value that follows the **“=”** symbol.
